# Technology-enabled primary eye health care in Pakistan

**Published:** 2022-06-07

**Authors:** Zahid Awan

**Affiliations:** 1 Inclusive Eye Health Projects Manager: CBM International, Islamabad, Pakistan.

**Mobile technology helped to optimise primary eye health care in Chakwal district, Pakistan, thereby increasing access to specialist eye health for those who need it.**

**Figure:**
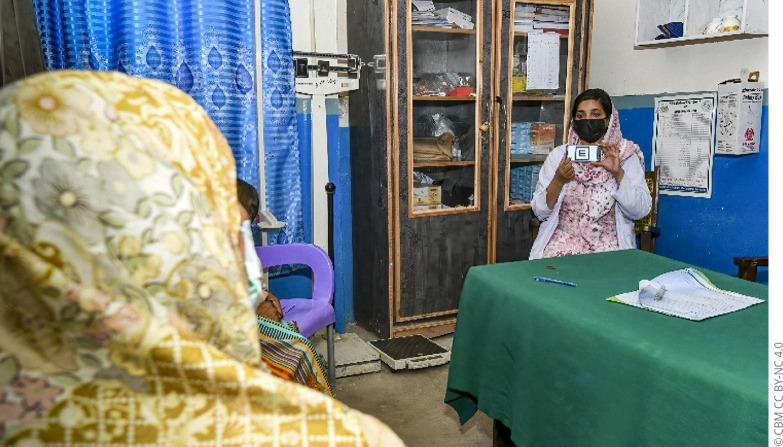
A lady health visitor tests visual acuity through Peek Acuity on an Android smartphone at a basic health unit in Chakwal district, Punjab. **PAKISTAN**

In 2018, the Pakistan National Committee for Eye Health, together with CBM and Peek Vision, launched a technology-enabled primary eye care project to assess, monitor, and improve eye care services in Chakwal district.

As previous studies in Pakistan have shown,^1^ referral pathways must be optimised in order to increase access to specialist eye health for patients. The CBM-Peek project aims to increase screening at primary health care level by including eye health promotion and awareness raising about available eye services in the community, thereby reducing pressure on secondary and tertiary eye care services.

**Figure 1:**
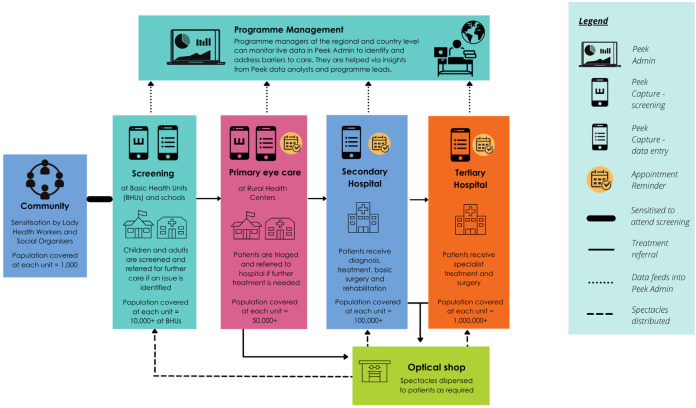
Referral pathways of the CBM and Peek Vision primary eye care project in Pakistan.

The project ensures that patients are connected to care using referral systems that link all levels of the eye health system (Figure 1). Real-time information about the whole programme is visible to the programme managers via the Peek Admin app, and the Peek Capture app is used at various stages to support visual acuity measurement, decision making, and to capture patient information.

## Programme structure

**Community level**

The project involves training Pakistan's lady health workers (community-based health care workers) in Chakwal district to do the following:

1. Create awareness in their catchment communities about the availability of eye care services at nearby facilities (known as ‘sensitisation’).
2. Identify and refer patients with eye conditions.
3. Follow patients up after referral or treatment.

Lady health workers are employed by the ministry of health, and each one is responsible for providing primary health care to approximately 1,000 people in their local or catchment community.

### Primary level health care: basic health units

Basic health units each serve a population of around 10,000 people. Around 110 of Pakistan's lady health visitors (health centre-based workers) in Chakwal District were trained to carry out vision testing using the Peek Capture app. The app incorporates Peek's clinically validated visual acuity test to identify individuals with vision impairment, and it also includes specially adapted questionnaires that support the lady health visitors to identify people with other eye conditions and refer them. School health and nutrition supervisors who are trained to use the Peek Capture app were also deployed in schools throughout the Chakwal district to screen and refer children identified with eye conditions or visual impairment to primary health care facilities.

### Primary level health care: rural health centres

Peek Capture is also used at rural health centres to screen walk-in patients. Optometrists see patients referred from the basic health units and any walk-in patients identified as needing eye care. The optometrists validate the visual acuity measured during screening (as a means of quality control), perform refraction, and prescribe spectacles to those who need them. They also conduct anterior and posterior chamber examinations and can refer patients to secondary or tertiary eye care services. Their actions, decisions, and referrals are recorded in the Peek Capture app.

## Secondary and tertiary health care

At the secondary and tertiary health care units, teams of ophthalmologists and ophthalmic allied personnel perform triage, treat referred cases, and provide training if required.

## Programme impact

**Figure 2:**
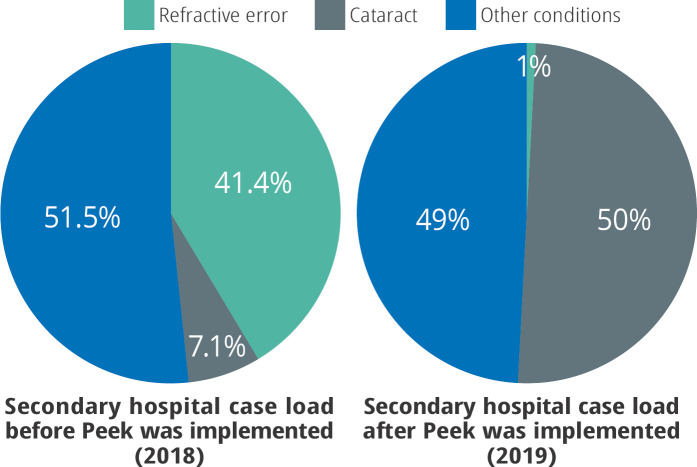
Secondary hospital case load before (2018) and after (2019) the project was implemented.

Before the programme was introduced, more than 41% of eye health consultations in hospitals were related to refractive errors, which can usually be treated outside the hospital by an optometrist. Since the start of the project, this proportion has been reduced to less than 1%. As a result, hospital eye health services are in a better position to treat patients with more complex eye conditions, such as cataract (Figure 2).

From November 2018 to the end of December, 2021:

- 79% of patients had their needs met at the primary level, 21% required referral to secondary care, and 1% required tertiary care.
- The number of people screened improved from 774 per month to almost 18,000 per month
- The percentage of false positive referrals from screening was reduced from 16.5% to 5.9%.

In addition, more people attended hospital within 30 days of their referral (12% in 2019, increasing to 66% in 2020). There was a greater increase in attendance of hospital appointments for women: from 45% in 2019 to 78% in 2020; for men this figure increased from 48% in 2019 to 70% in 2020.

## Next steps

Almost all the eye health facilities in Chakwal have now been connected using Peek Vision technology. The remaining facilities are due to be added to the system in 2022. We are also planning to expand this further, by equipping 1,500 lady health workers to conduct household-level screening and referral.

The data captured by the programme has also been used to advocate for eye health at the government level. As a result, the government of Pakistan has approved draft plans to strengthen eye care through digital solutions, and it has made a commitment to provide relevant human resources and help to expand the programme to other districts.

**Data collection and monitoring**

Thanks to the integrated system and dashboard, ‘live’ data is always available and accessible by the project's administrative and management team. Aspects covered include community screening, school screening, triage, referral, specialist referral, adherence to referrals, and spectacle prescription.

The data available on the Peek dashboard can be categorised and analysed by gender, age, location, project segment, and diagnostic conditions. It also enables the project staff members and field team to know the status of each step in the patient's journey, whether it is planned, pending, or completed.

As a result, gaps were highlighted so that services could be improved where needed and the quality of referrals could be improved.
